# Effectiveness of health literacy intervention on cardiovascular diseases among university students of Pakistan

**DOI:** 10.1186/s12913-019-4348-y

**Published:** 2019-07-19

**Authors:** Maria Nazar, Shahzad Ali Khan, Ramesh Kumar, Assad Hafeez

**Affiliations:** Health Services Academy, Islamabad, Pakistan

**Keywords:** Health literacy, College students, University students, Cardiovascular diseases, Heart attack, Symptom and stroke

## Abstract

**Background:**

Global burden of cardiovascular diseases is alarming which is intricately linked with health literacy. To what extent improvement in health literacy can lower down cardiovascular diseases occurrence has not yet properly documented. This study focused on assessing the knowledge and existing lifestyle behavior about cardiac diseases among university students. We further aimed to improve this awareness after imparting an educational intervention among undergraduate non-medical students to sensitize them about risk factors.

**Method:**

A pre and post approaches with cross sectional study design was conducted in University of Gujrat during April–September 2017. Using structured questionnaire comprising of response items about hypertension, heart attack, stroke and preventive practices, data was randomly collected from students (*n* = 100). Survey respondents were also given a lecture regarding cardiovascular diseases awareness and a post test evaluation was also conducted on same group of students.

**Results:**

With response rate of 86.95%, mean age of participating students was 21.2 (SD ± 1.34) years. Female students comprised of 53% out of which 57% were from rural background. Assessment of cardiovascular disease knowledge revealed maximum mean pre test score 30.53 (SD ± 7.61) and for post test 40.65 (SD ± 4.34) (*p* < 0.00). Mean score for using preventive practices was 13.02 (SD ± 2.97) for pre test whereas for post test it was 14.09 (SD ± 2.90) (*p* < 0.00). Intervention impact was significant on hypertension related complications (*p* < 0.000), symptoms of heart attack (p < 0.000), symptoms of stroke (p < 0.000) and preventive practices (p < 0.00).

**Conclusion:**

Findings presented here show a fair degree of awareness among university students about study title prior to any educational intervention. However, by attending educational session, a significant increase in the positive lifestyle behavior and knowledge was noticed. We conclude that health promotion activities in educational institutes to sensitize students can bring rational changes in Pakistani society to promote healthy behavior and minimize cardiovascular disease risks.

## Background

Health Literacy (HL) is the degree to which people are able to access, understand, appraise and communicate information to engage with demand of different health contexts in order to promote and maintain good health across the life course [1]. It has been repeatedly shown in different studies that health literacy in general is lower that related to the poor health outcomes in Pakistan [2–4]. About 17.9 million deaths reported due to cardiovascular diseases (CVD) in 2016, which contributes about 31% global mortality and major causes of death were heart attack and stroke [5]. However, the number of deaths would be increased by 23.3 million in next ten years [6]. Low and middle-income countries contribute nearly 82% in CVD related mortality due to their poor fragile health system and health illiteracy [5]. Hypertension is the major contributor of CVD and its complications are responsible for 9.4 million deaths every year [7]. Almost half of the deaths in Eastern Mediterranean Region (EMRO) occurs due to CVD results in major economic loss [8].

Pakistan reported 19% of total mortality due to CVD [9, 10]. Poor health literacy on prevention and control of CVD results in high mortality [3]. Hence, the health awareness and education plays a vital role in decreasing the burden of cardiovascular diseases. This would enhance the skills among patients to prevent and live better with cardiac problems and adopt their healthy lifestyles. Self-management of cardiac diseases could be learned through effective health education that improves their communication and self-confidence on management [3]. Though, this problem is prevailing in our masses but the younger population studying in colleges and universities might play a vital role by getting the proper information and strategies on management of cardiac diseases. They will play a vital role in further dissemination this information and helps in getting healthy behaviors their during early adulthood. Study recommends that the health education among youth could reverse the burden of cardiac mortality among general public [11]. To what extent awareness is needed to help people to transform their daily lifestyle practices and modifying their behavior is uncertain. Further studies are required in urban and rural areas of the country to engage people in self-management through health literacy (HL). In the context, a study was designed to assess level of CVD knowledge among non-medical students and evaluate the effectiveness of educational intervention by focusing behavioral factors that has potential to improve health literacy. We aimed to bring positive changes among young students through cost effective methods of communication and see whether increasing HL enables the students to become sensitive against CVD risk factors.

## Methods

### Study design

A cross sectional study design with pre and post intervention was conducted in 2017 by including undergraduate, non-medical students from only public university of Gujrat located in the Punjab province of Pakistan.

### Sampling and tool

A proportion sample size calculation formula was used to calculate the sample size of 100 students by adopting the stratified sampling technique based on their department and academic year of study. Overall 115 students were selected randomly from the enrollment registers and all were included in pretest. However, only 100 were successful to complete this intervention hence, the response rate was (87%). Two measurements, before and after was conducted by interviewing the students on adopted, pretested, piloted, validated tool [6, 12, 13]. Pretesting was conducted by interviewing ten university students from adjacent district.

### Data analysis

Descriptive statistics was performed for sociodemographic variables were analyzed by using SPSS version 23 for frequency and percentages and knowledge level was measured in two levels; yes and no. Variables on hypertension had 12 questions with score 0–29, heart attack symptoms had 8 question with score 0–8, stroke symptoms had 4 questions with score 0–8, health literacy had 8 questions with score 0–53, preventive practices variables had 8 questions scored from 5 to 22 (low to high). Frequencies with percentages were obtained for each question (pre and post) then mean scoring of pre test was compared to mean scoring of posttest of each question in each section and looked for statistical significance on paired t-test. Total mean score of each variable was obtained and compared with respective posttest mean score. In the last overall total knowledge’s mean score of pre and posttest was compared for statistical significance. Preventive practices were also analyzed in the same manner and compared for statistical significance.

### Intervention

Educational intervention consisted on 90 min session was given The content of session was on CVD risk factors (modifiable and non-modifiable), epidemiology of CVD, normal and high measurements of hypertension (HTN), its complications, medication, epidemiology and symptoms of heart attack, symptoms of stroke and preventive practices about knowing their usual BP, use of salt and fats intake, physical activity and its frequency, smoking habit of sample students and information seeking behavior. Baseline assessment was performed prior to start this intervention and end line data were collected after the intervention.

## Results

Baseline data was obtained from 115 students and 100 were finally completed the end assessment with response rate of (87%). Socio-demographic characteristics of the participants can be seen in Table 1. The mean age of students was (21, 1.34 ± SD) years, above half (53%) students were females out of which (57%) were residing in rural areas. Around (58%) fathers of the respondents were educate at least twelve years of schooling and their average income was 200–300 US $. Media was the dominant source of information regarding CVD followed by family/friends, teachers, doctors and other sources. The intervention had a positive significant effect (*p* = < 0.05) after the training on their knowledge in different variables presented in (Table 2). Overall mean score showed significant difference between pre and post-test scores. In baseline mean score obtained was (31, SD ± 7.6) which has increased to (41, SD ± 4.3) in post-test; the average change and intervention effect was 10. All the variables have improved after the intervention (Table 3).

**Table 1:** Socio-demographic characteristics of the participants (*n* = 100)

Variables		Frequency (%)
Gender	Male	47 (47)
	Female	53 (53)
Age	18–25 years	100 (100)
	Mean age	21
Place they belong	Urban	43 (43)
	Rural	57 (57)
Father’s Educational Level	Illiterate	2 (2)
	12 years schooling	58 (785
	Graduation	28 (28)
	Post-graduation	12 (12)
Father’s Monthly income	< 200 US $	44 (44)
	< 500 US $	34 (34)
	> 500 US $	22 (22)
Source of information on CVD	Media	45 (45)
	Family and friends	21 (21)
	Teachers	15 (15)
	Books, news and seminars	14 (14)
	Health Professional	5 (5)

**Table 2:** Participant’s Health information about cardiac diseases

Variables	Pre-test (%)	Post-test (%)	*p*-value
*Health knowledge on Blood Pressure*			
Normal BP is 130/80 mmHg	60	86	< 0.05
Normal BP is 160/100 mmHg	63	85	
High BP can cause stroke	57	84	
High BP can cause heart attack	75	90	
High BP can cause kidney problem	30	62	
High BP can cause eye problems	22	56	
BP usually lasts for long time	49	63	
Losing weight usually makes BP	58	74	
Eating less salt normalize BP	53	72	
High BP need medicine	45	56	
Symptoms in high BP	10	37	
High BP is dangerous	10	31	
*Information about cardiac attack symptoms*			
Sudden trouble	25	63	< 0.05
Symptoms are same in Male and female	59	82	
Feeling weak, light headed or faint	21	58	
Turning gray or pale	38	56	
Chest pain that radiate towards shoulder, jaw and arm	77	91	
Difficulty in breathing or shortness of breath	63	80	
Stress	10	60	
Diabetes	57	83	
Sudden trouble in speaking and confusion	46	59	
Sudden dizziness, trouble walking and loss of balance	57	72	
Sudden weakness or numbness of face, arm or leg	43	80	
Sudden trouble seeing in one or both eyes	24	68	
*Health practices related variables*			
Check your usual Blood pressure	41	55	< 0.05
Add salt to your meals	67	48	
Avoid eating fat rich food	33	30	
Physical activity	74	78	
Smoking	16	16	> 0.05
Body weight (BMI) is appropriate	33	42	< 0.05
Interested to know about CVD	48	53

**Table 3:** Positive significant change after the intervention

Sections	Maximum score	Pre-test	Post-test	Absolute Change	*p*-Value
Hypertension & its complications	29	17.080	21.38	4.30	< 0.000
Symptoms of Heart attack	16	9.280	12.92	3.68	< 0.000
Symptoms of Stroke	08	4.170	6.35	2.180	< 0.000
Total Score (HL of CVD)	53	30.53	40.65	10.12	< 0.000
Preventive practices	22	13.02	14.09	1.07	< 0.000

## Discussion

Health literacy is an important intervention in protecting public from chronic diseases; hence poor HL among the community would results significant morbidity, mortality and other health complications [3]. This study has clearly shown that mortality and morbidity by CVD can be reduced by the effective awareness intervention. Varying HL levels found in our survey group manifest a large number of CVD risk factors that could treated if diagnosed earlier. Hence, this study has highlighted the value of HL awareness campaign among university students. Areas where access to health facilities is limited, the high priority to increase HL would become the only solution to promote health [3].

Findings of this study showed considerably low level of awareness about hypertension related information and symptoms of stroke that believed HL intervention is required in specific direction. Specific intervention for the raising HL among students showed a significant changes after posttest assessment. As we assessed from our study, the knowledge of participants raised after a simple educational intervention. A substantial improvement was observed in questions about symptoms of stroke, complications of HTN on kidneys and eyes, HTN as a silent killer and taking stress as most important cause of heart attack which further confirms that educated community such as university students or people with college education or higher can be used as targeted community groups for specific health intervention. In some studies, interventions in terms of information and training showed positive change in cardiovascular parameters in different age groups. Study supported to our findings with remarkable changes in knowledge level, attitudes and behavior of school boys regarding health physical activities which contributes heart attack prevention [14]. Several other reports have demonstrated that interactive lectures and informative session with students could bring considerable improvement in health literacy [15]. Other study launched a holistic approached intervention to improve physical activity, environment and healthy food for three years and claimed that Body Mass Index (BMI) and fitness has considerably improved among school students [16]. Pilot study on individuals with a positive family history of CVD desired results have been depicted by improved and healthy lifestyle among respondents after delivering informative lectures [17]. A positive impact of health education intervention was achieved in a study appropriate communication methods can increase the impact of HE and prognosis of disease. Core abilities were developed in students and teachers about HL to empower their health [18]. A school based study revealed that a single, simple and least costly educational intervention can enhance preventive strategies among young adolescents and identified the best time to intervene have not yet started [19]. A randomized intervention study highlighted that counseling on screening and emphasis on lifestyle modifications had long lasting effects on physical activity [20].

Participants get sensitized to know their blood pressure in posttest despite the recent guidelines recommended that everyone above the age of 18 years should checked for HTN and after 20 years should be investigated for cholesterol levels as a preventive measure [21]. Pakistan Demographic Health Survey (PDHS) reported prevalence of obesity was 11% in rural men and 19% in rural women but in urban areas it was 23 and 40% in men and women respectively [22]. Recent research work showed that more reduction in dietary salt intake lowered morbidity and mortality by CVD [23].

Findings of this study have clearly indicated that students got sensitized about various CVD indicators. Across the globe, studies prove poor HL regarding CVD among populations which was subsequently improved by health interventions [15, 16, 19]. This study was a minor step to fill the knowledge gap among students, but there is a constant need to promote health literacy in every section of community to get better health outcomes. It is recommended that the university administration and academicians should include information related to cardiac health in their curriculum for to provide health literacy to their students. Time and funding constraints were the major limitation in our study.

## Conclusions

The undergraduate students were initially lacking the ability to recognize the symptoms of stroke and hypertension. However, upon attending the education session, their health literacy were significantly increased..

## Data Availability

Most data generated during this study are included in this manuscript. Other data that may support the findings of this research are available from the corresponding author on request.

## Abbreviations

BMI: Body Mass Index
CVD: Cardiovascular diseases
EMRO: Eastern Mediterranean Region
HL: Health Literacy
HTN: Hypertension
PDHS: Pakistan Demographic Health Survey
